# Comparative Effectiveness of Emergency Resuscitative Thoracotomy versus Closed Chest Compressions among Patients with Critical Blunt Trauma: A Nationwide Cohort Study in Japan

**DOI:** 10.1371/journal.pone.0145963

**Published:** 2016-01-14

**Authors:** Kodai Suzuki, Shigeaki Inoue, Seiji Morita, Nobuo Watanabe, Ayumi Shintani, Sadaki Inokuchi, Shinji Ogura

**Affiliations:** 1 Department of Emergency and Disaster Medicine, Gifu University Graduate School of Medicine, Gifu, Japan; 2 Department of Emergency and Critical Care Medicine, Tokai University School of Medicine, Isehara, Kanagawa, Japan; 3 Department of Clinical Epidemiology and Biostatistics, Osaka University Graduate School of Medicine, Suita, Osaka, Japan; University of Florida, UNITED STATES

## Abstract

**Background:**

Although emergency resuscitative thoracotomy is performed as a salvage maneuver for critical blunt trauma patients, evidence supporting superior effectiveness of emergency resuscitative thoracotomy compared to conventional closed-chest compressions remains insufficient. The objective of this study was to investigate whether emergency resuscitative thoracotomy at the emergency department or in the operating room was associated with favourable outcomes after blunt trauma and to compare its effectiveness with that of closed-chest compressions.

**Methods:**

This was a retrospective nationwide cohort study. Data were obtained from the Japan Trauma Data Bank for the period between 2004 and 2012. The primary and secondary outcomes were patient survival rates 24 h and 28 d after emergency department arrival. Statistical analyses were performed using multivariable generalized mixed-effects regression analysis. We adjusted for the effects of different hospitals by introducing random intercepts in regression analysis to account for the differential quality of emergency resuscitative thoracotomy at hospitals where patients in cardiac arrest were treated. Sensitivity analyses were performed using propensity score matching.

**Results:**

In total, 1,377 consecutive, critical blunt trauma patients who received cardiopulmonary resuscitation in the emergency department or operating room were included in the study. Of these patients, 484 (35.1%) underwent emergency resuscitative thoracotomy and 893 (64.9%) received closed-chest compressions. Compared to closed-chest compressions, emergency resuscitative thoracotomy was associated with lower survival rate 24 h after emergency department arrival (4.5% vs. 17.5%, respectively, *P* < 0.001) and 28 d after arrival (1.2% vs. 6.0%, respectively, *P* < 0.001). Multivariable generalized mixed-effects regression analysis with and without a propensity score-matched dataset revealed that the odds ratio for an unfavorable survival rate after 24 h was lower for emergency resuscitative thoracotomy than for closed-chest compressions (*P* < 0.001).

**Conclusions:**

Emergency resuscitative thoracotomy was independently associated with decreased odds of a favorable survival rate compared to closed-chest compressions.

## Introduction

Trauma is one of the most notable and leading causes of death in all age groups, particularly among children, adolescents, and young adults [1]. Despite improvements in pre-hospital transport and management of trauma victims [2–4], the annual death toll still exceeds 5,800,000 worldwide [5]. Severe trauma often leads to cardiac arrest and a low survival rate (5.6%; 0%–17%) [6].

Thoracic trauma caused by penetrating or blunt trauma accounts for 25%–50% of all casualties [7] and 50% of casualties among patients with civilian trauma [8]. More than 50% of cases involving trauma-induced cardiac arrest result from blunt trauma [6], which leads to higher mortality than penetrating trauma [6]. Blunt thoracic trauma frequently occurs as a result of rapid deceleration or crushing in traffic accidents followed by massive hemothorax, great vessel disruption, pulmonary contusion, and cardiac injury [9]. These multiple and complex mechanisms of injury contribute to higher mortality in critical blunt trauma patients.

Emergency resuscitative thoracotomy (ERT) is performed as a salvage maneuver for selected patients in extremis or with cardiac arrest shortly after emergency department (ED) arrival [10]. The goals of ERT include pericardial tamponade release, intrathoracic vascular and/or cardiac hemostasis, massive air embolism or bronchopleural fistula control and management, open-chest cardiopulmonary resuscitation, and temporary descending thoracic aorta occlusion [11–13]. Some reports have demonstrated better survival rates after ERT among patients with penetrating trauma than among those with blunt trauma [14–17]. However, appropriate resuscitative maneuvers for critical blunt trauma patients remain unclear because of the lack of published information on the effectiveness of ERT in such patients. Therefore, the overall aim of this study was to test the hypothesis that ERT is associated with favorable outcomes among critical blunt trauma patients by evaluating its effectiveness compared to that of manual closed-chest compressions (CCC), a conventional method of cardiopulmonary resuscitation [18], using clinical data collected from multiple hospitals registered in the nationwide Japan Trauma Data Bank (JTDB) (https://www.jtcr-jatec.org/traumabank/dataroom/ethics_intro.htm).

## Materials and Methods

### Study design and participants

The JTDB is a Japanese trauma registry organization established by the Trauma Registry Committee of the Japanese Association for the Surgery of Trauma and the Committee for Clinical Care Evaluation in the Japanese Association for Acute Medicine. This study was approved by the Institutional Review Board for Clinical Research of Gifu University. Trauma patients were registered in the JTDB upon admission to any of the 221 hospitals included in the database. No consent was required because the data were extracted from the registry and analyzed anonymously.

In this study, cardiac arrest was defined as the inability to detect the blood pressure of a patient upon ED arrival and the failure to detect any palpable arteries. In the registry, blood pressure was recorded as 40 mmHg if the pulse was palpable, even if the actual pressure was undetectable, and 0 mmHg if the pulse was not palpable. The indications for ERT were extremis or cardiac arrest shortly after ED arrival. ERT was defined as thoracotomy conducted within 24 h of ED arrival. The ERT group consisted of all patients who underwent ERT regardless of receiving prior CCC, while the CCC group consisted of patients who only received CCC during resuscitation.

**Fig 1:**
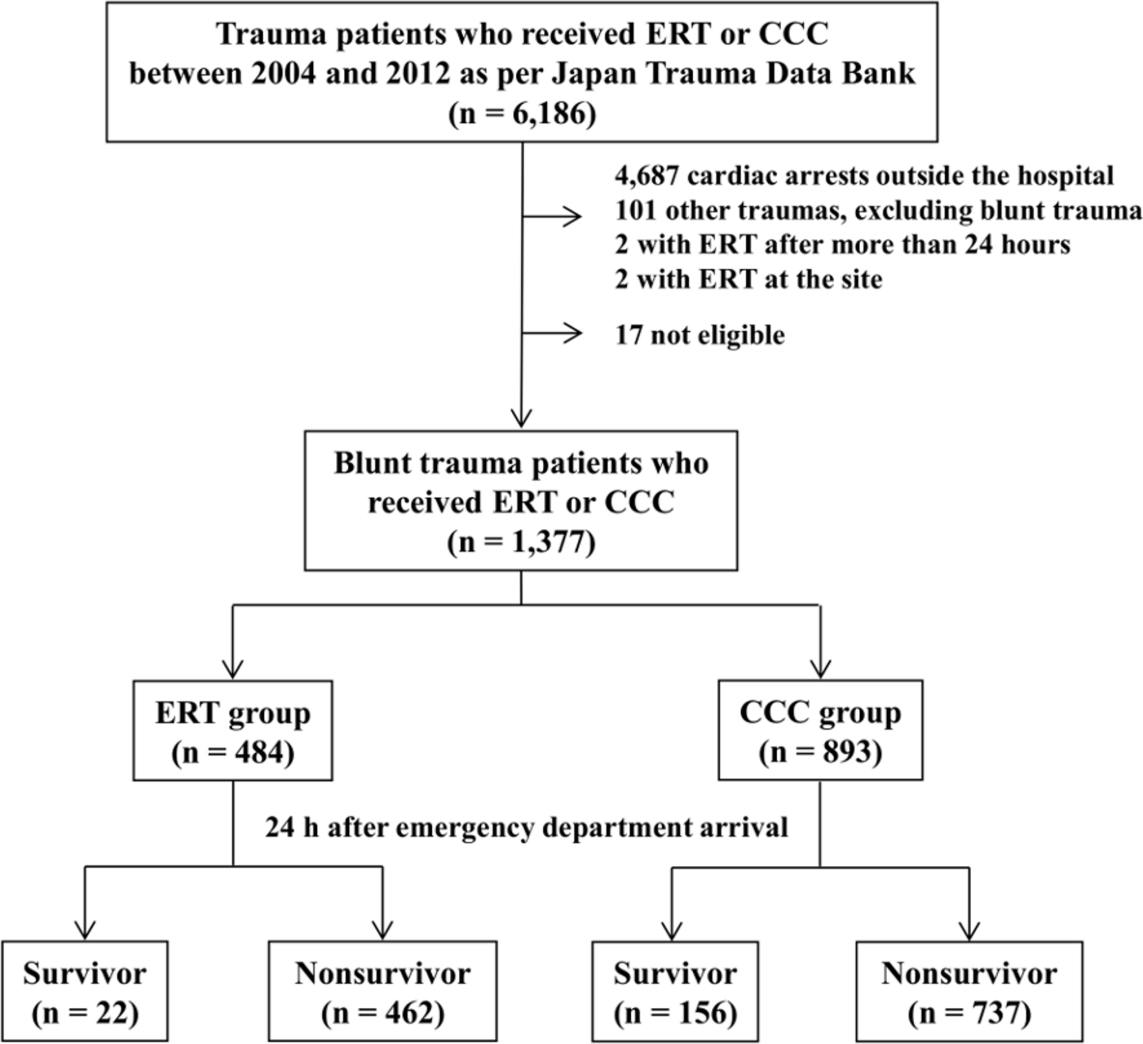
Flow chart of patients included in the study. In total, 1,377 patients were enrolled from the Japan Trauma Data Bank between 2004 and 2012. Emergency resuscitative thoracotomy (ERT): 484. Closed-chest compressions (CCC): 895. Survivors for > 24 h after emergency department arrival: ERT group, 22; CCC group, 156.

A total of 123,462 trauma patients were registered in the JTDB between January 2004 and December 2012. Of these patients, 6,188 who underwent ERT or received CCC at the ED or in the operating room were included in the study. Among these patients, data were excluded for those: (a) with cardiac arrest and loss of signs of life on ED arrival, (b) without blunt trauma, (c) who underwent ERT more than 24 h after ED arrival, (d) who underwent ERT at the accident site, and (e) those with incomplete data. Seventeen patients were excluded because of insufficient data regarding outcome (Fig 1). The data were analyzed for the remaining 1,377 blunt trauma patients who underwent ERT or received CCC (Fig 1).

### Study endpoints

The primary outcome was the 24-h survival rate, defined as survival for > 24 h after ED arrival. The secondary outcome was the 28-d survival rate after ED arrival, defined as survival for > 28 d after ED arrival.

### Statistical analyses

To assess the independent effects of ERT compared with those of CCC, it was important to adjust for factors associated with a likelihood of undergoing ERT and mortality. Regression adjustment was used for the primary analysis, where potential confounders (covariates) were simultaneously included with the ERT or CCC variables in multivariable generalized mixed-effects regression analysis. Covariates were selected *a priori* based on factors associated with mortality within a permissible number computed using the 10 events per variable rule. We adjusted for the effects of different hospitals by introducing random intercepts in regression analysis to account for the differential quality of ERT at hospitals where the patients who went into cardiac arrest were treated.

We also conducted sensitivity analyses using propensity score matching (PSM), where the propensity score was computed as the probability of undergoing ERT as a function of potential confounders selected *a priori* on the basis of biological plausibility and *a priori* knowledge. The variables included age, sex, onset year, transfer process, transporter, cause of trauma, vital signs on ED arrival (i.e., systolic blood pressure, respiratory rate, heart rate, Glasgow Coma Scale score, and temperature), injury severity score (ISS), the rate of positive focused assessment with sonography for trauma results, and blood transfusion within 24 h of ED arrival. The choice of these factors was discussed with trauma surgeons, and the factors were then included as covariates in a logistic regression model with either ERT or CCC as the dependent variable.

PSM was performed with one-to-one matching using a caliper with 0.25 standard deviations of the linear propensity score, resulting in a sample size of 852. The data were analyzed using IBM SPSS statistic 22 (IBM SPSS, Chicago, IL, U.S.A.) and R version 3.0.1 (www.r-project.org). The data are presented as the means ± standard deviations. Differences between the ERT and CCC groups were analyzed using the Mann–Whitney U-test for nonparametric data or the Chi-square test. Statistical significance was set at a two-sided *P* < 0.05. Finally, we used multiple imputation in a logistic regression model to compute the propensity score to handle missing values at the time of modeling using predictive mean matching [19].

## Results

### Patient characteristics

A total of 1,377 patients who were registered in the JTDB between 2004 and 2012 were analyzed in this study. These included 484 patients in the ERT group and 893 in the CCC group (Fig 1). The patient demographics were compared between both groups (Table 1) and between the 24-h survivors and nonsurvivors (S1 Table).

**Table 1:** Characteristics of critical blunt trauma patients who received emergency resuscitative thoracotomy or closed-chest compressions.

		CPR method	
Variable	Total (n = 1377)	ERT (n = 484)	CCC (n = 893)	*P*-value
**Age, y (mean ± SD), n (%)**	57 ± 23	53 ± 22	59 ± 23	<0.001
0–17	52 (4)	14 (3)	39 (4)	
18–64	704 (51)	295 (61)	409 (46)	
65<	615 (45)	172 (36)	443 (50)	
Missing	5 (0)	3 (1)	2 (0)	
**Male, n (%)**	921 (67)	345 (71)	576 (65)	0.011
**Onset year, n (%)**				0.21
2004–2006	205 (15)	61 (13)	144 (16)	
2007–2009	493 (36)	179 (37)	314 (35)	
2010–2012	679 (49)	244 (50)	435 (49)	
**Cause of trauma, n (%)**				0.559
Accident	1,034 (75)	354 (73)	680 (76)	
Self-inflicted injury (suicide)	199 (14)	78 (16)	121 (14)	
Injury	6 (0)	2 (0)	4 (0)	
Workplace accident	82 (6)	31 (6)	51 (6)	
Missing	56 (4)	19 (4)	37 (4)	
**Transfer mode, n (%)**				0.229
Transported directly from the injury site	1,235 (90)	433 (90)	802 (90)	
Transferred from other hospital	108 (8)	40 (8)	68 (8)	
Ambulance except	10 (1)	1 (0)	9 (1)	
Missing	24 (2)	10 (2)	14 (2)	
**Transport type, n (%)**				0.555
Ambulance	1,095 (80)	383 (79)	712 (80)	
Ambulance with physician	77 (6)	24 (5)	53 (6)	
Private automobile	6 (0)	1 (0)	5 (0)	
Air ambulance	170 (12)	67 (14)	103 (12)	
Missing	29 (2)	9 (2)	20 (2)	
**Pre-hospital CPR, n (%)**	144 (10.5)	38 (7.9)	106 (11.8)	0.021
**Vital signs on ED arrival (mean ± SD)**				
Systolic blood pressure, mmHg	86 ± 42	76 ± 37	91 ± 43	<0.001
<90	812 (59)	334 (69)	478 (54)	
≧90	565 (41)	150 (31)	415 (47)	
Heart rate, beats/min	101 ± 35	106 ± 37	99 ± 34	<0.001
<60	148 (11)	54 (11)	94 (11)	
60–99	421 (31)	111 (23)	310 (35)	
≥100	744 (54)	298 (62)	446 (50)	
Missing	64 (5)	21 (4)	43 (5)	
Respiratory rate, breaths/min	22 ± 14	24 ± 14	21 ± 13	<0.001
<10	206 (15)	74 (15)	132 (15)	
10–29	557 (40)	165 (34)	392 (44)	
≥30	384 (28)	180 (37)	204 (23)	
Missing	230 (17)	65 (13)	165 (18)	
Glasgow Coma Scale	6.8±4.4	7.1 ± 4.3	6.6 ± 4.4	0.053
Missing	71 (5)	17 (4)	54 (6)	
Temperature, °C	35.4 ± 1.5	35.3 ± 1.5	35.5 ± 1.5	0.238
Missing	483 (35)	183 (38)	300 (34)	
**AIS (mean ± SD)**				
1 (Head)	3.8 ± 1.3	3.5 ± 1.3	3.9 ± 1.3	<0.001
2 (Face)	1.7 ± 1.1	1.6 ± 1.1	1.7 ± 1.1	0.915
3 (Neck)	2.8 ± 2.2	1.8 ± 1.5	3.0 ± 2.3	0.224
4 (Thorax)	2.9 ± 2.1	3.6 ± 1.9	2.5 ± 2.1	<0.001
5 (Abdomen and Pelvic Contents)	3.6 ± 1.6	3.8 ± 1.5	3.5 ± 1.7	0.003
6 (Cervical Spine)	2.9 ± 1.4	2.6 ± 1.2	3.1 ± 1.5	0.019
7 (Upper Extremity)	2.1 ± 0.7	2.2 ± 0.6	2.0 ± 0.8	0.021
8 (Lower Extremity)	3.4 ± 1.3	3.5 ± 1.2	3.4 ± 1.3	0.171
9 (External)	1.1 ± 0.2	1.1 ± 0.3	1.0 ± 0.2	0.326
**ISS (mean ± SD)**	36.1 ± 15.8	38.4 ± 15.3	34.9 ± 16.0	<0.001
0–24	239 (17)	64 (13)	175 (20)	
25–44	736 (53)	256 (53)	480 (54)	
45<	334 (24)	139 (29)	195 (22)	
Missing	68 (5)	25 (5)	43 (5)	
**FAST, n (%)**				<0.001
Positive	470 (34)	252 (52)	218 (24)	
Negative	717 (52)	184 (38)	533 (60)	
Not conducted	122 (9)	33 (7)	89 (10)	
Missing	68 (5)	15 (3)	53 (6)	
**Blood transfusion, n (%)**				<0.001
Transfusion	918 (67)	409 (85)	509 (57)	
No transfusion	434 (32)	66 (14)	368 (41)	
Missing	25 (2)	9 (2)	16 (2)	
**IABO, n (%)**	184 (13.4)	85 (17.6)	99 (11.1)	0.001
**REBOA, n (%)**	221 (16.0)	212 (43.8)	9 (1.0)	<0.001
**IABO or REBOA, n (%)**	370 (26.9)	265 (54.8)	105 (11.8)	<0.001
**Drinking, n (%)**	57 (4)	17 (4)	40 (4)	0.002
Missing	655 (48)	262 (54)	393 (44)	
**Survival, n (%)**				
24 h	178 (13)	22 (5)	156 (17)	<0.001
24-h survivors who underwent pre-hospital CPR, n (%)	31 (21.5)	1 (2.6)	30 (28.3)	0.001
24-h survivors who underwent IABO or REBOA, n (%)	16 (1.1)	10 (2.1)	5 (0.6)	<0.001
28 d	60 (4)	6 (1)	54 (6)	<0.001
Survival to discharge	93 (7)	9 (2)	84 (9)	<0.001

ERT: Emergency resuscitative thoracotomy

CCC: Closed-chest compressions

CPR: Cardiopulmonary resuscitation

SD: Standard deviation

ED: Emergency department

FAST: Focused assessment with sonography for trauma

AIS: Abbreviated injury scale

ISS: Injury severity score

IABO: Intra-aortic balloon occlusion

REBOA: Resuscitative endovascular balloon occlusion of the aorta

The average age and mean systolic blood pressure were significantly lower in the ERT group than in the CCC group, while the mean respiratory rate and heart rate were significantly higher in the ERT group than in the CCC group (*P* < 0.001 for all, Table 1). The abbreviated injury scale (AIS) score for the thorax, and the abdomen and pelvis was significantly higher for the ERT group than for the CCC group (*P* < 0.001 and *P* = 0.003, Table 1). The rates of positive focused assessment with sonography for trauma results and the mean ISS were significantly higher in the ERT group than in the CCC group (*P* < 0.001 for both, Table 1). The number of patients who underwent resuscitative endovascular balloon occlusion of the aorta or intra-aortic balloon occlusion was significantly higher in the ERT group than in the CCC group (*P* < 0.001, Table 1). The 24-h and 28-d survival rates were significantly lower in the ERT group than in the CCC group (24-h survival, 4.5% with ERT vs. 17.5% with CCC, *P* < 0.001; 28-d survival, 1.2% with ERT vs. 6.0% with CCC, *P* < 0.001; Table 1). The proportion of patients transported by air to the ED, systolic blood pressure, and the Glasgow Coma Scale score were significantly higher, while the AIS score for the thorax, and abdomen and pelvis was significantly lower among the survivors 24 h after ED arrival than among the nonsurvivors (*P* < 0.05 for all, S1 Table). PSM revealed a well-balanced ERT (n = 371) and CCC (n = 371) cohort, with no significant differences between groups in any parameter except the 24-h survival rate and onset year (Table 2). Onset year was included because it was unbalanced even in the matched cohort.

**Table 2:** Characteristics of the matched cohorts of critical blunt trauma patients who received emergency resuscitative thoracotomy or closed-chest compressions.

	CPR method			
Variable	ERT (n = 371)	CCC (n = 371)	Combined (n = 742)	*P*-value
**Age, y (mean ± SD)**	55 ± 22	55 ± 24	54 ± 23	0.43
**Male, n (%)**	252 (68)	249 (67)	501 (68)	0.81
**Onset year, n (%)**				0.034
2004–2006	60 (16)	53 (14)	113 (15)	
2007–2009	165 (44)	137 (37)	302 (41)	
2010–2012	146 (39)	181 (49)	327 (44)	
**Cause of trauma, n (%)**				0.79
Accident	278 (78)	280 (79)	558 (79)	
Self-inflicted injury (suicide)	56 (16)	58 (16)	114 (16)	
Other	21 (6)	17 (5)	57 (7)	
**Transfer mode, n (%)**				0.79
Transported directly from the injury site	327 (88)	332 (89)	659 (89)	
Transferred from another hospital	33 (9)	30 (8)	63 (8)	
Ambulance except	1 (0)	2 (1)	3 (0)	
Missing	10 (3)	7 (2)	17 (2)	
**Transport type, n (%)**				0.77
Ambulance	289 (80)	294 (79)	583 (81)	
Air ambulance	55 (15)	48 (13)	103 (14)	
Other	18 (5)	18 (5)	36 (5)	
**Vital signs on ED arrival (mean ± SD)**				
Systolic blood pressure, mmHg	79 ± 37	78 ± 36	78 ± 36	0.78
Heart rate, beats/min	106 ± 37	103 ± 35	104 ± 36	0.25
Respiratory rate, breaths/min	24 ± 13	23 ± 13	23 ± 13	0.15
Glasgow Coma Scale	7.2 ± 4.4	6.9 ± 4.5	7.1 ± 4.4	0.35
Temperature, °C	35.4 ± 1.4	35.3 ± 1.7	35.3 ± 1.6	0.65
**ISS (mean ± SD)**	38 ± 16	38 ± 16	38 ± 16	0.73
**FAST, n (%)**				
Positive	168 (50)	152 (49)	320 (49)	0.74
**Blood transfusion, n (%)**				
Transfusion	301 (83)	293 (80)	594 (82)	0.36
**Survival, n (%)**				
24 h	352 (95)	326 (88)	678 (91)	<0.001

ERT: Emergency resuscitative thoracotomy

CCC: Closed-chest compressions

CPR: Cardiopulmonary resuscitation

SD: Standard deviation

ED: Emergency department

FAST: Focused assessment with sonography for trauma

AIS: Abbreviated injury scale

ISS: Injury severity score

### Multivariable generalized mixed-effects regression analysis for 24-h survival

According to multivariable generalized mixed-effects regression analysis, the odds ratio (OR) for survival 24 h after ED arrival was significantly lower for the ERT group than for the CCC group [OR, 3.78; 95% confidence interval (CI), 1.77–8.08; *P* < 0.001; Table 3]. Analysis with a propensity score-matched dataset showed a similar result (OR, 2.83; 95% CI, 1.57–5.12; *P* < 0.001; Table 3).

**Table 3:** Analysis of the effects of emergency resuscitative thoracotomy vs. closed-chest compressions after adjusting for potential confounders.

	OR	95% CI	*P*-value
**Multivariable generalized mixed-effects regression analysis**			
with adjustment of covariate	3.78	1.77–8.08	< 0.001
with propensity score-matched dataset	2.83	1.57–5.12	< 0.001

ERT: Emergency resuscitative thoracotomy

CCC: Closed-chest compressions

OR: Odds ratio

CI: Confidence interval

### Subgroup analysis

S2 Table shows the data for the 22 ERT survivors 24 h after ED arrival. Patients who were discharged to home or to other hospitals (n = 10) tended to have higher respiratory and heart rates, and a lower ISS compared with those who later died in the hospital (n = 9).

## Discussion

In this nationwide Japanese observational study of critical blunt trauma patients, the patients who underwent ERT had a lower survival rate compared to those who received CCC. Furthermore, ERT was independently associated with decreased odds of a favorable survival rate compared with CCC. It has been reported that the survival rate after ERT was lower among blunt trauma patients than those with penetrating trauma [14–17]. However, to the best of our knowledge, this is the first report showing that ERT is associated with a reduced possibility of survival compared with CCC in critical blunt trauma patients, after adjustment using several types of statistical methods.

ERT is an established procedure for the treatment of life-threatening chest injuries [20–22]. The goals of ERT include pericardial tamponade or tension pneumothorax release, intrathoracic hemorrhage and massive air embolism or bronchopleural fistula control and management, open cardiac massage, and cross-clamping of the thoracic aorta, thus restoring and maintaining perfusion to the heart and brain and preventing additional blood loss from distal hemorrhage sites [11–13, 23]. However, the use of ERT for critically injured patients has remained debatable since its inception in the 1960s [10]. ERT is a useful technique for the resuscitation of patients with penetrating trauma who are in extremis, particularly those with penetrating thoracic trauma or cardiac injuries [16]. In patients presenting with vital signs after a penetrating thoracic injury, the survival rate after ERT may be as high as 38% [11].

Large retrospective studies over the past two decades have revealed a decreased survival rate after ERT for blunt trauma patients (0% to 6%) compared to those of patients with penetrating trauma [14, 24–30]. In a large retrospective meta-analysis based on 25 years of published data, Rhee et al. described 24 studies that included a total of 4,620 ERT cases [16]. These data showed an overall survival rate of 7.4%, with normal neurological outcomes in 92.4% survivors. Furthermore, the survival rate was higher for patients with penetrating trauma (8.8%), particularly cardiac injuries (19.4%) and stab wounds (16.8%), than for those with blunt trauma (1.4%) [16]. The American College of Surgeons’ Committee on Trauma reviewed 42 studies that included 7,035 ERT cases in its *Practice Management Guidelines for Emergency Department Thoracotomy*. These data showed an overall survival rate of 7.8% and survival rates of 11.2% and 1.6% for patients with penetrating and blunt trauma, respectively [17]. Therefore, the eighth edition of the *Advanced Trauma Life Support* guidelines provides specific recommendations for performing ERT in the setting of penetrating thoracic trauma with detectable electrical activity, but not in the setting of blunt trauma with electrical cardiac activity in a patient without a detectable pulse [31].

Despite abundant evidence for the ineffectiveness of ERT in blunt trauma patients (and a negative statement regarding the ineffectiveness that is included in most guidelines), ERT is still performed. Passos et al. reported that 51% of the ERTs performed on 123 patients were considered inappropriate, which resulted in substantial expenses, a waste of resources, an increased risk of exposure of health-care workers to possible blood-borne infections, and no survival benefits [32–33]. Brown et al. reported that ERT was cost-effective for penetrating trauma, but not for blunt trauma considering the reported survival rate and risk of neurological impairment [34]. Moreover, the prevalence of human immunodeficiency virus (HIV) seropositivity in the trauma patient population is reportedly 24%, with an even higher prevalence of hepatitis [35]. Taken together, the total additional cost incurred from accidental viral exposure associated with thoracotomy was $1,377, with a high probability of HIV and chronic hepatitis C seroconversion [33].

Since Kouwenhoven et al. first reported the effectiveness of CCC over open cardiac massage for cardiac arrest in 1960 [36], it has been administered as a conventional resuscitation maneuver for cardiac arrest and was recommended by the American Heart Association [37]. Our data demonstrate that CCC results in a higher survival rate compared with ERT in critical blunt trauma patients, indicating that CCC may be more beneficial than ERT as a resuscitative maneuver in these patients. However, it was difficult to interpret the survival differences shown in Table 1 because there were several significant differences among the populations analyzed. Therefore, to further confirm the results, multivariable generalized mixed-effects regression analysis, logistic regression with multiple imputation, and multivariable analysis of the original cohort using inverse probability weighting were performed. All of the analyses showed that CCC resulted in better outcomes than ERT (data not shown).

This study has several limitations. First, there was no information regarding the optimal time for performing ERT in critical patients in the JTDB. This is an important issue because the effectiveness of ERT for trauma patients depends on the time from cardiac arrest to ERT [38]. In a previous study, the time from loss of pulse to thoracotomy was significantly shorter in the survivor group [39]. This is biologically reasonable and is supported by some evidence in the literature; indeed, better outcomes were observed in patients who underwent ERT within 30 minutes of injury than in patients who underwent ERT after 30 minutes [40]. However, the patients selected in this study showed signs of life on arrival. Therefore, we believe there may have been more patients who received excessive CCC for a prolonged duration before receiving ERT. Second, there was no information regarding the length of extremis, signs of life prior to ERT, the time of cardiac arrest or resumption of spontaneous circulation, and the presence or absence of bystander resuscitation in the JTDB. There was also no information regarding neurologic outcomes with sustained CCC or the overall disposition of the patients in the JTDB. Finally, unmeasured confounders were not considered because this was a retrospective nationwide cohort study and the collection of more detailed data would have been difficult. Thus, the observed results may have resulted in unmeasured confounding. Despite these limitations, this study demonstrates the limited efficacy of ERT in critical blunt trauma patients.

## Conclusions

ERT was independently associated with decreased odds of a favorable survival rate compared with CCC in critical blunt trauma patients. The criteria for performing ERT for the treatment of blunt trauma must be reconsidered.

## Supporting Information

S1 Table Characteristics of critical patients who received emergency resuscitative thoracotomy or closed-chest compression after sustaining blunt trauma. (XLSX)

S2 Table Characteristics of 24-h survivors who received emergency resuscitative thoracotomy after sustaining blunt trauma. (XLSX)
